# Effects of Pharmacological Inhibitors of NADPH Oxidase on Myogenic Contractility and Evoked Vasoactive Responses in Rat Resistance Arteries

**DOI:** 10.3389/fphys.2021.752366

**Published:** 2022-01-24

**Authors:** Dylan J. Kendrick, Ramesh C. Mishra, Cini Mathew John, Hai-Lei Zhu, Andrew P. Braun

**Affiliations:** Department of Physiology and Pharmacology, Cumming School of Medicine and Libin Cardiovascular Institute, University of Calgary, Calgary, AB, Canada

**Keywords:** myogenic activity, vasodilation, resistance artery, endothelium, NOX

## Abstract

Reactive oxygen species (ROS), such as superoxide anions and hydrogen peroxide, are reported to contribute to the dynamic regulation of contractility in various arterial preparations, however, the situation in pressurized, myogenically active resistance arteries is much less clear. In the present study, we have utilized established pharmacological inhibitors of NADPH oxidase activity to examine the potential contribution of ROS to intrinsic myogenic contractility in adult Sprague–Dawley rat resistance arteries and responses to vasoactive agents acting *via* the endothelium (i.e., acetylcholine, SKA-31) or smooth muscle (i.e., sodium nitroprusside, phenylephrine). In cannulated and pressurized cremaster skeletal muscle and middle cerebral arteries, the NOX inhibitors 2-acetylphenothiazine (2-APT) and VAS2870, selective for NOX1 and NOX2, respectively, evoked concentration-dependent inhibition of basal myogenic tone in a reversible and irreversible manner, respectively, whereas the non-selective inhibitor apocynin augmented myogenic contractility. The vasodilatory actions of 2-APT and VAS2870 occurred primarily *via* the vascular endothelium and smooth muscle, respectively. Functional responses to established endothelium-dependent and –independent vasoactive agents were largely unaltered in the presence of either 2-APT or apocynin. In cremaster arteries from Type 2 Diabetic (T2D) Goto-Kakizaki rats with endothelial dysfunction, treatment with either 2-APT or apocynin did not modify stimulus-evoked vasoactive responses, but did affect basal myogenic tone. These same NOX inhibitors produced robust inhibition of total NADPH oxidase activity in aortic tissue homogenates from control and T2D rats, and NOX isozymes 1, 2 and 4, along with superoxide dismutase 1, were detected by qPCR in cremaster arteries and aorta from both species. Based on the diverse effects that we observed for established, chemically distinct NOX inhibitors, the functional contribution of vascular NADPH oxidase activity to stimulus-evoked vasoactive signaling in myogenically active, small resistance arteries remains unclear.

## Introduction

Reactive oxygen species (ROS) can have deleterious effects on cellular activity, but it is also apparent that these molecules contribute to cell signaling events (Thannickal and Fanburg, 2000). Under physiological conditions, superoxide anions are generated as a normal by-product of tissue metabolism (i.e., oxidative phosphorylation), which are then converted rapidly to hydrogen peroxide (H_2_O_2_) *via* superoxide dismutase (Wolin, 2000). In the vascular wall, another prominent source of ROS is the enzymatic activity of NADPH oxidase, which generates superoxide anions that are often considered harmful to cellular function. Indeed, multiple studies have implicated NADPH oxidase activity in the pathogenesis of vascular dysfunction and cardiovascular disease (Lassegue et al., 2012). For example, superoxide anions interfere with endothelium-dependent dilation by combining with nitric oxide (NO) to form peroxynitrite, a reactive by-product that reduces NO bioavailability and chemically modifies cellular constituents, such as lipids, DNA and proteins, thereby promoting tissue injury (Pacher et al., 2007).

Arterial contractility is dynamically governed by both chemical and mechanical signaling, as well as intrinsic processes within the vascular wall that generate myogenic tone (Davis and Hill, 1999; Félétou et al., 2011; Davis, 2012; Vanhoutte et al., 2017). H_2_O_2_ is reported to relax a variety of arterial preparations (Matoba et al., 2000, 2002; Miura et al., 2003; Thengchaisri and Kuo, 2003; Cseko et al., 2004; Li et al., 2016) and the vascular endothelium is a recognized source of H_2_O_2_ in the vasculature (Babbs et al., 1992; Skepper et al., 1998). Under pathophysiological conditions that compromise NO bioavailability, H_2_O_2_ may act as a “substitute” vasodilatory factor in small arteries (Liu et al., 2003, 2011; Miura et al., 2003; Ellinsworth et al., 2015; Gutterman et al., 2016). Mechanistically, H_2_O_2_ is reported to hyperpolarize smooth muscle (Beny and von der Weid, 1991), stimulate large conductance, Ca^2+^-activated K^+^ (BK_*Ca*_) channels (Barlow and White, 1998; Barlow et al., 2000), and activate cGMP-dependent protein kinase (PKG) *via* cysteine oxidation (Burgoyne et al., 2007; Dou et al., 2012; Zhang et al., 2012; Sheehe et al., 2018), which are all known to promote vasodilation. Khavandi et al. (2016) have further reported that elevated intraluminal pressure in resistance arteries may stimulate H_2_O_2_ production and PKG activation as an intrinsic feedback mechanism to regulate vascular tone.

Pharmacological inhibition of NADPH oxidase activity has been employed extensively as a strategy to interrogate the acute actions of superoxide anions and ROS such as H_2_O_2_ in the vasculature, and despite the considerable literature in this area, there is very limited information describing the actions of such compounds in intact, myogenically active resistance arteries under physiological pressures. In the present study, we have examined how acute pharmacological inhibition of intrinsic NADPH oxidase activity may impact basal contractility, along with endothelium-dependent and –independent vasoactive responsiveness in myogenically active arteries from healthy rats and Type 2 Diabetic rats exhibiting endothelial dysfunction/reduced NO bioavailability. We anticipated that pharmacological inhibition of NADPH oxidase enzymes in the vascular wall using established blockers (i.e., apocynin, a pan-NOX inhibitor, 2-acetylphenothiazine (aka ML171), a selective inhibitor for NOX1 and VAS2870, a NOX2 selective inhibitor) would restrict acute ROS (i.e., superoxide and H_2_O_2_) generation and signaling, thereby altering vasoactive responsiveness.

## Materials and Methods

All procedures for animal treatment and care have been approved by the University of Calgary Animal Care Committee (Research protocol # AC20-0109) and coincide with the guidelines for the care and use of laboratory animals established by the Canadian Council on Animal Care. Male Sprague–Dawley rats (18–22 weeks of age) were obtained from Charles River Laboratories (Laval, PQ, Canada) and housed under standard conditions (i.e., 12 h light/dark cycle, 22°C) with free access to food and water. Type 2 Diabetic male Goto-Kakizaki rats (20–24 weeks of age) were purchased from Taconic Biosciences (Germantown, NY, United States) and housed under similar conditions.

### Arterial Pressure Myography

This procedure was carried out as previously described (Mishra et al., 2015, 2018). Rats were injected intraperitoneally (IP) with sodium pentobarbital (50 mg/kg) to induce surgical anesthesia and overdose. The cremaster muscles were then surgically removed and transferred to a cooled (4°C) dissection bath containing Krebs’ buffer (115 NaCl, 5 mM KCl, 25 mM NaHCO_3_, 1.2 mM MgCl_2_, 2.0 mM CaCl_2_, 1.2 mM KH_2_PO_4_ and 10 mM D-glucose), with the pH adjusted to 7.4. Cremaster 1A and 2A arteries (150–220 μm maximal intraluminal diameter) were carefully isolated and cannulated on glass pipettes that had been fitted into a pressure myography chamber (Living Systems, Burlington, VT, United States). To isolate middle cerebral arteries (∼150–250 μm maximal intraluminal diameter), rats were decapitated following anesthesia, and the brain was removed and placed in the cooled dissection chamber. Glass cannulae with tapered ends were pulled from borosilicate capillary tubes (0.7 mm ID and 1.2 mm OD) using a Sutter P-97 pipette puller; tips were then manually broken to generate tip openings in the range of 40–60 microns. The vessel lumen was filled with Krebs’ solution containing 1% bovine serum albumin (BSA), as described by Duling (Duling et al., 1981). The chamber was transferred to the stage of an inverted microscope and the bath was perfused with Krebs’ buffer at 7 mL/min using a peristaltic pump and suction line. Bath solution was gassed with 95% air/5% CO_2_ and temperature was maintained at ∼34°C for cremaster arteries and ∼37°C for cerebral arteries. Intraluminal pressure was increased in a stepwise manner under no flow conditions from 0 mmHg and sustained at 70 mmHg using a pressure column, allowing 5-min of equilibration at each pressure. Arteries that failed to develop > 20% myogenic tone within 20–30 min were discarded. Intraluminal vessel diameter (i.e., inner surface of the arterial wall) was measured throughout the protocol at a sampling frequency of 1 Hz using an automated diameter tracking system (IonOptix, Milton, MA, United States).

For experiments involving intraluminal perfusion of middle cerebral arteries, a combined pressure servo-controller and servo-pump system was employed (Living Systems Instrumentation, Burlington, VT, United States). Intraluminal flow (∼25 μl/min) was initiated as arteries were being pressurized to develop myogenic tone, and was maintained throughout the remainder of the experimental protocol. This level of intraluminal flow generated an average shear stress of 48.3 ± 15.9 dyn/cm^2^ (mean ± SD, *n* = 3) as calculated from the equation:

Shear stress (τ) = 4ηQ/πr^3^, where η = fluid viscosity (0.008 dyn s/cm^2^), Q = intraluminal flow in ml/s and r = arterial radius in cm (Kuo et al., 1995). The average vessel radius in myogenically constricted arteries in the presence of intraluminal flow was 43.2 ± 5.3 microns (mean ± SD, *n* = 3).

The tip diameters of inflow and outflow cannulae used for intraluminal perfusion were matched as closely as possible for a given artery to achieve uniform intraluminal flow along the vessel length. Cannulae tips typically ranged in size from ∼40–60 μm, and were selected based upon the diameter of the isolated artery at the time of mounting. As the tips of mounting cannulae were typically smaller than the lumens of pressurized vessels, this difference will contribute to flow resistance through the arterial lumen. 2-APT was perfused through the vessel lumen during the experiment by switching the inflow cannula line from the control buffer to one containing 10 μM 2-APT, followed by return to the control buffer for 2-APT washout.

Maximal inhibition of developed myogenic tone by 2-APT and VAS2870 in cremaster and middle cerebral arteries and associated IC_50_ values (see Figures 2, 3) were calculated from non-linear regression fits of the data to the Hill equation as follows (Mishra et al., 2015):


Drug-evokedinhibitionofmyogenictone=Rmax/(1+IC/50[drug])N

Where Rmax = the calculated maximal degree of drug-evoked inhibition, IC_50_ is the calculated drug concentration producing half-maximal inhibition of the maximal response, [drug] represents the concentrations of drug used experimentally, and *N* = the calculated Hill coefficient.

### NADPH-Oxidase Activity Assay

NADPH oxidase activity was measured in tissue homogenates by a luminescence assay using NADPH as the primary substrate and lucigenin as an acceptor for oxidase generated superoxide molecules, as modified from Griendling et al. (1994). Experimentally, freshly isolated tissue was homogenized (5–10% w/v) using a small probe polytron in ice-cold buffer containing (final): 50 mM Tris–HCl, pH 7.5, 150 mM NaCl, 1 mM EGTA, 1 mM EDTA, 1 mM benzamidine, 1 mM PMSF, and 5 μg/ml each of leupeptin, pepstatin A and aprotinin. The crude homogenate was filtered through two layers of cotton gauze and the protein concentration was determined using a modified Lowry assay kit (Bio-Rad Laboratories). Typically, 100 μg of homogenate protein was aliquoted per tube and each experimental condition was assayed in triplicate. Tissue homogenate + NADPH (1 mM final) was pre-incubated at 37°C for 2 min, and the reaction was started by addition of lucigenin (50 μM final); total assay volume was 0.1 ml with a final homogenate protein concentration of 1 mg/ml. For the background control condition, tissue homogenate was replaced by homogenization buffer alone. When present, individual NOX inhibitors were incubated with tissue homogenates on ice for ∼30 min prior to the start of the assay. Lucigenin oxidation leads to photon emission, which was measured for 0.4 s at 0.1 Hz over a 5 min period using a Monolight 3010 luminometer. Recently, Rezende et al. (2016, 2017) have raised concerns that this assay may also detect cytochrome P450 activity in cellular homogenates.

### Quantitative PCR

Following isolation and removal of surrounding fat and connective tissue, total RNA was extracted from cremaster arteries and the aorta using the RNeasy micro kit (Qiagen) according to the manufacturer’s instructions. Total RNA from liver tissue was extracted to act as a positive control, with PCR wells minus the addition of cDNA acting as the negative, non-template control. The concentration and quality of extracted RNA were determined using a Nanodrop instrument. Complementary DNA was prepared from total RNA using a Quantinova synthesis kit (Qiagen) and quantitative RT-PCR was performed using the Kapa SYBR Fast Universal quantitative PCR (qPCR) kit (Kapa Biosystems) and validated primer sets obtained from Integrated DNA Technologies (see Table 1). The molecular targets within the tissues were NAPDH oxidase isozymes 1–4, along with superoxide dismutase 1 (SOD1); rat glyceraldehyde 3-phosphate dehydrogenase (GAPDH) was used as the internal reference gene. Control reactions and those containing cDNA from cremaster arteries and the aorta were performed with 1 ng of template per reaction organized in a PCR well plate. The heating protocol per cycle consisted of 95°C for 5 s, 55°C for 10 s and 72°C for 10 s using an Eppendorf Realplex 4 Master cycler and was extended to 40 cycles. PCR specificity was examined by dissociation curve analysis. Assay validation was confirmed by testing serial dilutions of pooled template cDNAs, indicating a linear dynamic range of 2.8–0.0028 ng of template, which yielded percent efficiencies ranging from 100–118%. Control samples lacking template yielded no detectable fluorescence. Note that NOX3 mRNA could not be detected reproducibly in vascular tissue following 40 cycles of amplification, which prevented reliable quantification. This finding is consistent with the reported minimal expression of NOX3 isoform in cardiovascular tissues (Bedard and Krause, 2007; Lassegue et al., 2012). Expression of target genes in tissue-derived RNA samples was determined using the relative expression software tool (REST) version 2.0.13 (Pfaffl et al., 2002). Our reporting of qPCR data follows the published MIQE guidelines (Bustin et al., 2009).

**TABLE 1:** Forward and reverse primers used in qPCR experiments.

	Forward primer	Reverse primer
NOX 1	5′-TCAGGAAGACGTACACAAACAG-3′	5′-GCTCCAGACCTCCATTTGAC-3′
NOX 2	5′-ACCTCCATCCTGAATCCCTT-3′	5′-GTCCCATGTTCCTGTATCTGT-3′
NOX 3	5′-TTTGTCTAGTTGCCGTCTCC-3′	5′-TGCTAATTCTGTTACCTGTCAGTC-3′
NOX 4	5′-AGAAGCTGTAACCATGAGGAAC-3′	5′-CAGCAAGATACCAGAATGAGGAT-3′
SOD	5′-GCCTTGTGTATTGTCCCCATA-3′	5′-CGTCATTCACTTCGAGCAGA-3′
GAPDH	5′-GTAACCAGGCGTCCGATAC-3′	5′-TCTCTGCTCCTCCCTGTTC-3′

### Data Analysis

Results are presented as mean ± S.D; *N* = the number of animals used experimentally in a given protocol. Statistical analyses were carried out using either SigmaPlot 14 or GraphPad Prism 8 software. A Student’s *t*-test was utilized for analysis of one or two sample groups; a one-way ANOVA with a Newman–Keuls *post hoc* test was used for comparisons among 3 or more groups.

## Results

### Exogenous H_2_O_2_ Evokes Concentration-Dependent Dilation of Myogenically Constricted Cremaster Arteries

**FIGURE 1:**
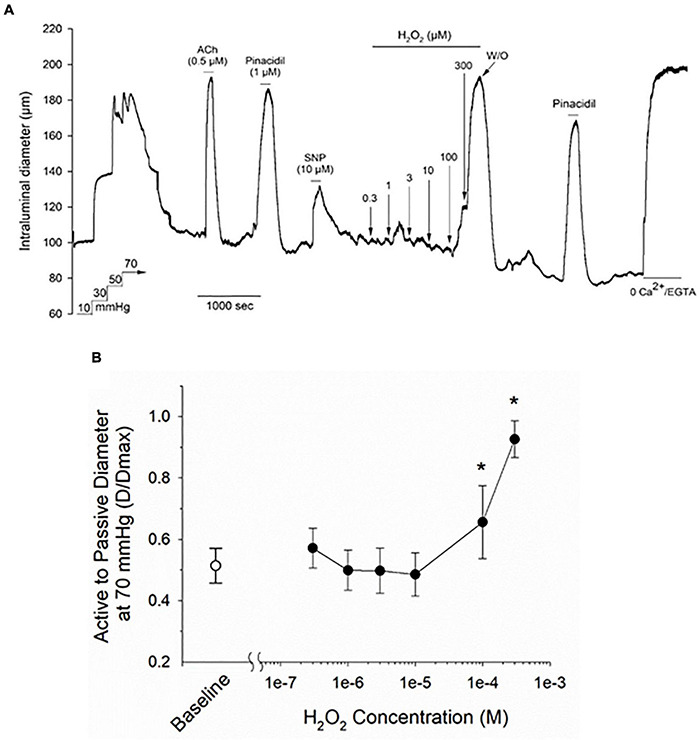
Treatment with hydrogen peroxide (H_2_O_2_) evokes a vasodilatory response in myogenically contracted rat cremaster arteries. Panel **(A)** shows a representative tracing from a cannulated cremaster artery from an adult Sprague–Dawley rat exposed to a range of H_2_O_2_ concentrations, along with endothelium-dependent and -independent vasoactive agents. All agents were added to the bath perfusion solution and treatments are indicated by the horizontal lines above and below the tracing. Maximal arterial diameter was determined at the end of the protocol by bath perfusion with a nominally Ca^2+^ free physiological saline solution (i.e., 0 added CaCl_2_ + 2 mM EGTA). The ratios of intraluminal diameter in myogenically active arteries vs. diameter under passive conditions (i.e., 0 added CaCl_2_ + 2 mM EGTA) at baseline and in the presence of the indicated concentrations of H_2_O_2_ are plotted in panel **(B)**. The asterisk denotes a H_2_O_2_ evoked change that is significantly different from the baseline level, as determined by one-way ANOVA and a Newman–Keuls *post hoc* test (*P* < 0.05). Data are expressed as means ± SD, *n* = 5 animals.

Isolated and cannulated cremaster skeletal muscle 1A and 2A resistance arteries from adult Sprague–Dawley rats developed robust myogenic tone when pressurized to 70 mmHg (i.e., % decrease in maximal intraluminal diameter = 51.6 ± 7.4%, mean ± SD, *n* = 18). As illustrated by the representative tracing in Figure 1A, bath addition of H_2_O_2_ to myogenically constricted cremaster arteries produced a robust and reversible vasodilation that developed at concentrations above 10 μM, as quantified in Figure 1B. These myogenically active arteries also exhibited reversible vasodilations to acetylcholine (ACh), the smooth muscle K-ATP channel opener pinacidil and sodium nitroprusside (SNP), demonstrating intact endothelial and smooth muscle vasorelaxant signaling pathways in these vessels. Treatment of vessels with a bath solution containing nominally free CaCl_2_ + 2 mM EGTA at the end of the stimulation protocol was used to determine the maximal passive diameter at 70 mmHg intraluminal pressure.

### The Selective NOX Inhibitors 2-Acetylphenothiazine and VAS2870 Produce Concentration Dependent Inhibition of Basal Myogenic Tone in Rat Cremaster Arteries

**FIGURE 2:**
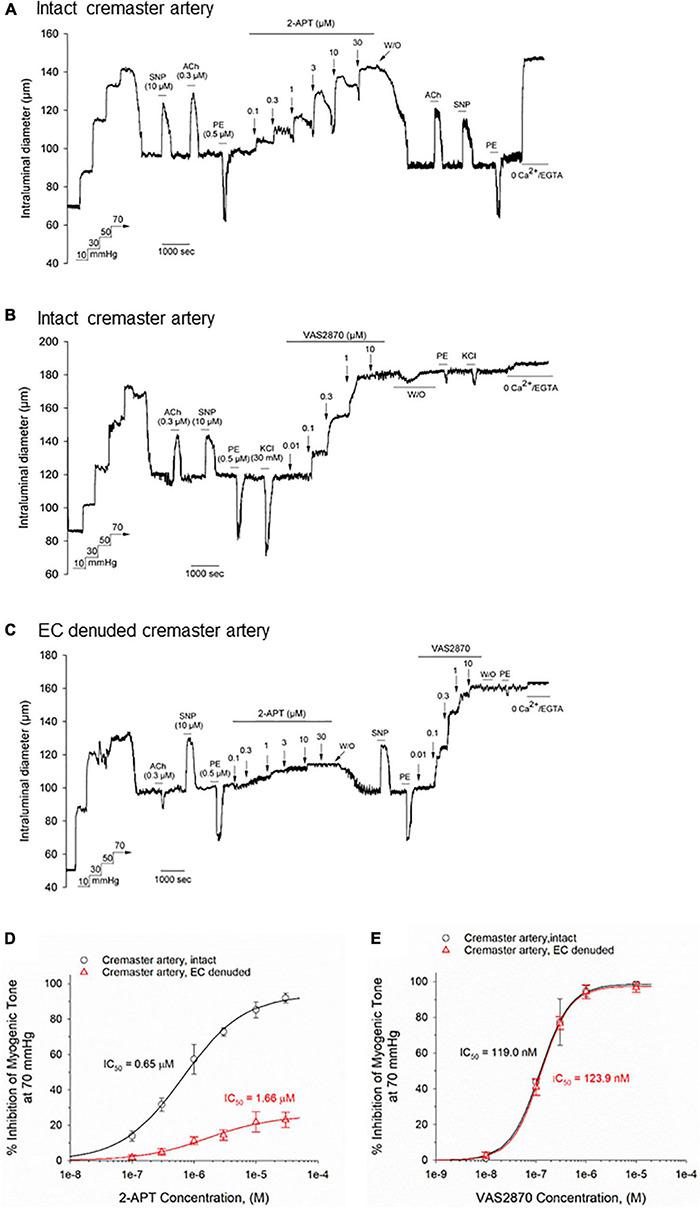
The selective NOX inhibitors 2-acetylphenothiazine (2-APT) and VAS2870 produce endothelium-dependent and -independent inhibition of basal myogenic tone in Sprague–Dawley rat cremaster arteries. Panel **(A)** shows a representative tracing of the reversible vasoactive responses to increasing concentrations of 2-APT (0.1–30 μM) perfused through the bath in a sequential manner, followed by washout. Stimulation of the artery with acetylcholine (ACh, 0.3 μM), sodium nitroprusside (SNP, 10 μM) and phenylephrine (PE, 0.5 μM) demonstrate responsiveness to endothelium-dependent and –independent vasoactive agents prior to and following 2-APT treatment. At the end of the experimental protocol, the artery was superfused with a physiological saline solution containing 0 added CaCl_2_ + 2 mM EGTA to evoke the maximal arterial diameter. The representative tracing in panel **(B)** displays a comparable experimental procedure as shown in panel **(A)**, in which the artery was treated with increasing concentrations of VAS2870. In panel **(C)**, an endothelium-denuded cremaster artery was treated with increasing concentrations of 2-APT, followed by washout and addition of increasing concentrations of VAS2870. Stimulation of arteries with ACh, SNP and PE was used to assess endothelium- and smooth muscle-dependent vasoactive responses. The horizontal lines above and below the tracings indicate the presence of a given stimulus or treatment. Panel **(D)** quantifies the concentration-dependent inhibition of myogenic tone at 70 mmHg by 2-APT for intact (black symbols and lines, *n* = 5) and endothelium-denuded arteries (red symbols and lines, *n* = 3 arteries). The plot in panel **(E)** quantifies the concentration-dependent inhibition of myogenic tone by VAS2870 for intact and endothelium-denuded cremaster arteries (*n* = 3 for each preparation). In both panels **(D,E)**, the indicated IC_50_ values for inhibition of myogenic tone and the non-linear regression fits to the plotted data points were calculated using a Hill equation (see section “Materials and Methods”). Data are presented as means ± S.D.

The observed sensitivity of myogenically constricted cremaster arteries to H_2_O_2_ raised the possibility that H_2_O_2_ may serve as an endogenous signaling molecule for vasodilatory stimuli acting on the vascular endothelium and/or smooth muscle. As NADPH oxidase activity represents an important source of superoxide anions used for H_2_O_2_ production, we asked whether inhibition of vascular NOX enzymes by the reported pharmacological blockers 2-acetylphenothiazine (2-APT, aka ML171), VAS2870 and apocynin (Selemidis et al., 2008; Altenhöfer et al., 2015) would modify endothelium-dependent vasodilation in myogenically constricted cremaster arteries. We first investigated whether these inhibitors exerted potential direct effects on myogenic contraction in cremaster arteries, and utilized vasoactive agents acting *via* vascular endothelium (i.e., ACh) or smooth muscle (i.e., SNP, phenylephrine) to confirm the responsiveness of actively constricted vessels. Unexpectedly, bath addition of the selective NOX1 inhibitor 2-APT to cremaster arteries produced a robust, concentration-dependent vasodilation (i.e., inhibition of myogenic tone) that decayed in magnitude over the ∼8 min application period and readily reversed upon drug washout (Figure 2A). Exposure to 2-APT did not impair subsequent vasoactive responses to ACh, SNP or phenylephrine (PE). We further observed that addition of the selective NOX2 inhibitor VAS2870 to intact cremaster arteries also produced a potent, concentration-dependent inhibition of myogenic contraction (Figure 2B). However, the direct vasoactive effect of VAS2870 did not readily reverse upon washout, and we observed impaired contractile responses to both PE and 30 mM KCl in VAS2870 treated arteries (Figure 2B). The magnitude and potency of 2-APT and VAS2870-evoked inhibition of myogenic tone in intact cremaster arteries are quantified in Figures 2D,E, respectively. To examine the potential contribution of the endothelium to these actions of 2-APT and VAS2870, the endothelial layer was denuded in a subset of cremaster arteries by passing an air bubble through the lumen of cannulated vessels prior to pressurization. Denuded arteries still developed robust myogenic tone, exhibited normal vasoactive responses to the smooth muscle agents SNP and PE, but no longer dilated in response to ACh (Figure 2C). Endothelial denudation significantly blunted the magnitude and potency of 2-APT-evoked vasodilation, without affecting the recovery of myogenic contraction following 2-APT washout. These data demonstrate that the vascular endothelium plays a major role in the vasodilatory action of 2-APT in cremaster arteries, and that this agent can also directly affect vascular smooth muscle. Endothelial denudation did not diminish either the magnitude or potency of VAS2870-mediated inhibition of steady-state myogenic tone (Figure 2E), nor did it alter the irreversibility of this effect. This observation demonstrates that VAS2870 acts directly on smooth muscle in cremaster arteries.

### 2-APT and VAS2870 Produce Concentration Dependent Inhibition of Basal Myogenic Tone in Rat Middle Cerebral Arteries

**FIGURE 3:**
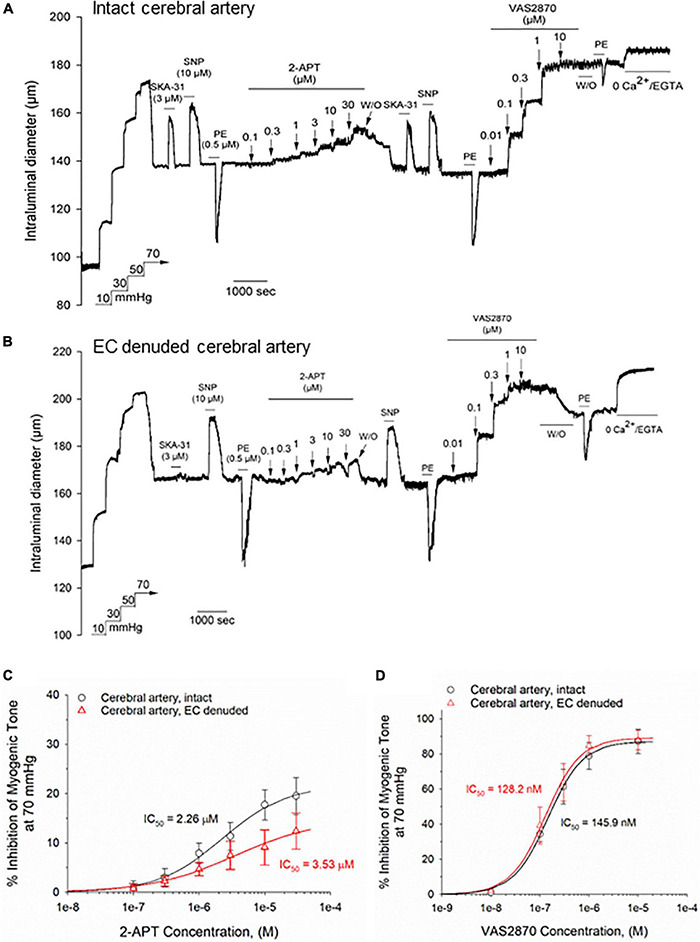
The NOX inhibitors 2-APT and VAS2870 inhibit developed myogenic tone in Sprague–Dawley rat cerebral arteries. Panel **(A)** shows a representative tracing of a myogenically contracted middle cerebral artery with intact endothelium, and reversible responses of the artery to the endothelium-dependent vasodilator SKA-31 (3 μM), the smooth muscle vasorelaxant sodium nitroprusside (SNP, 10 μM) and smooth muscle contractile agent phenylephrine (PE, 0.5 μM). Treatment of the artery with increasing concentrations of 2-APT evoked vasodilatory responses that reversed upon washout of 2-APT from the bath. VAS2870 (0.01–10 μM) was then perfused through the bath in a sequential manner, followed by washout. At the end of the protocol, PE stimulation was repeated, followed by perfusion of the bath with a physiological saline solution containing 0 added CaCl_2_ + 2 mM EGTA to evoke maximal arterial diameter. The horizontal lines above and below the tracing indicate the presence of the indicated stimulus or treatment. The representative tracing in panel **(B)** illustrates the effects of a very similar treatment protocol performed in an endothelial cell (EC)-denuded cerebral artery. The plots in panels **(C,D)** quantify the concentration-dependent inhibition of basal myogenic tone by 2-APT and VAS2870, respectively, for intact (*n* = 4) and EC-denuded cerebral arteries (*n* = 4–5). The indicated IC_50_ values for inhibition of myogenic tone and the non-linear regression fits to the plotted data symbols were calculated using a Hill equation (see section “Materials and Methods”). Data are presented as means ± S.D.

To determine if the observed vasodilatory effects of 2-APT and VAS2870 are selective for skeletal muscle resistance arteries, we examined these agents in myogenically active, middle cerebral arteries from adult male Sprague–Dawley rats. Miller et al. (2005) have reported that middle cerebral arteries from male Sprague–Dawley rats generate 10-fold higher levels of superoxide anion compared with peripheral arteries, suggesting that inhibition of NOX activity in these vessels may have a more noticeable effect on vasoactive responsiveness. The representative tracing in Figure 3A shows that the active diameter of intact, myogenically active, middle cerebral arteries was 27.3 ± 4.8% (*n* = 4) less than their maximal passive diameter at 70 mmHg intraluminal pressure and exhibited normal responses to endothelium-dependent and –independent vasoactive agents, as we have previously reported (Mishra et al., 2015). Bath exposure to 2-APT produced a reversible, concentration-dependent inhibition of myogenic tone in intact middle cerebral arteries (Figure 3A) that was reduced in magnitude and potency, and was comparable to data observed in endothelium-denuded cremaster arteries (compare average data in Figures 2D, 3C). Interestingly, intraluminal application of 2-APT in intact cerebral arteries evoked a twofold greater vasodilatory response compared with bath application of 2-APT in the same vessels (Supplementary Figure 1).

In middle cerebral arteries denuded of their endothelium (confirmed by loss of dilatory response to SKA-31), the concentration-dependent vasodilatory response to 2-APT was qualitatively similar to that observed in intact arteries, although the magnitude of effect was slightly blunted (Figure 3B). In contrast to the observed effects of 2-APT, bath application of VAS2870 to intact middle cerebral arteries produced a robust and largely irreversible inhibition of myogenic tone that was similar in magnitude and potency to that observed in intact cremaster arteries (compare Figures 3A,D with Figures 2B,E). In endothelium-denuded cerebral arteries, the magnitude and potency of VAS2870 evoked vasodilation were similar to those observed in intact vessels (Figures 3B,D). Following washout of VAS2870 from the bath, we observed that the PE-induced vasoconstrictor response was severely blunted, similar to observations in cremaster arteries (Figures 2B,C). In myogenically contracted, cerebral arteries denuded of endothelium, active intraluminal diameter was 23.6 ± 3.9% (*n* = 5) less than their maximal passive diameter at 70 mmHg intraluminal pressure.

### NOX Inhibitors Produce Concentration-Dependent Inhibition of NADPH Oxidase Activity in Rat Aortic Tissue Homogenates

To confirm that 2-APT and VAS2870 act as *bona fide* inhibitors of NADPH oxidase activity in vascular tissue, we examined the ability of these agents to inhibit enzymatic activity in aortic tissue homogenates from adult male Sprague–Dawley rats. Using an optimized *in vitro* biochemical assay to measure *de novo* superoxide anion generation, we independently verified that these agents directly inhibit NADPH oxidase activity at concentrations comparable to those reported for inhibition of NOX isozymes in other cell types (see Table 2) (Selemidis et al., 2008; Altenhöfer et al., 2015). To complement these biochemical data, we carried out q-PCR analysis and identified mRNA encoding NOX 1, 2 and 4 in rat cremaster arteries and aorta (Figure 8). Robust expression of mRNA for SOD 1, an enzyme responsible for the conversion of superoxide to H_2_O_2_, was also detected in both cremaster arteries and aorta.

**TABLE 2:** Inhibition of NADPH-oxidase (NOX) enzyme activity by apocynin, 2-APT and VAS2870 in Sprague–Dawley rat aortic homogenates.

Assay conditions	RLU (mean ± S.D.)	% Inhibition
Blank (NADPH + Lucigenin + Buffer)	161 ± 202	−
Homogenate + Lucigenin + PBS	187 ± 246	−
Homogenate + NADPH + Lucigenin (total)	31,236 ± 9,359	−
Homogenate + N + L + Apocynin (10 μM)	15,830 ± 3,404	49.3%
Homogenate + N + L + Apocynin (100 μM)	3,672 ± 1,528	88.2%
Homogenate + N + L + VAS2870 (0.01 μM)	28,270 ± 8,131	9.5%
Homogenate + N + L + VAS2870 (0.1 μM)	23,567 ± 5,028	24.6%
Homogenate + N + L + VAS2870 (0.3 μM)	10,754 ± 873	65.6%
Homogenate + N + L + VAS2870 (10 μM)	3,812 ± 849	87.8%
Homogenate + N + L + 2-APT (0.3 μM)	13,502 ± 3,729	56.8%
Homogenate + N + L + 2-APT (1 μM)	9,471 ± 1,043	69.7%
Homogenate + N + L + 2-APT (3 μM)	4,356 ± 997	86.1%

*NOX activity was evaluated by detection of superoxide radicals using a lucigenin-based reporter assay, as described in section “Materials and Methods.” Aliquots of aortic tissue homogenate were incubated for at least 30 min with either vehicle or the indicated concentrations of each NOX inhibitor before lucigenin was added to start the reaction. The percentage inhibition of superoxide generation was calculated as follows: [(Total NOX activity – NOX activity in presence of inhibitor)/total activity] × 100. The condition for total NOX activity = Homogenate + NADPH + Lucigenin. All assay conditions noted below were tested in triplicate for each tissue homogenate and readings were averaged. Values are presented as mean relative light units (RLU) ± S.D. For measurements of total NOX activity, 7 separate aortic tissue homogenates were utilized; for assays containing NOX inhibitors, n = 3–4 separate homogenates per condition. 2-APT, 2-acteylphenothiazine.*

### Treatment With the Selective NOX1 Inhibitor 2-APT Minimally Affects Vasoactive Responsiveness in Cremaster Arteries

**FIGURE 4:**
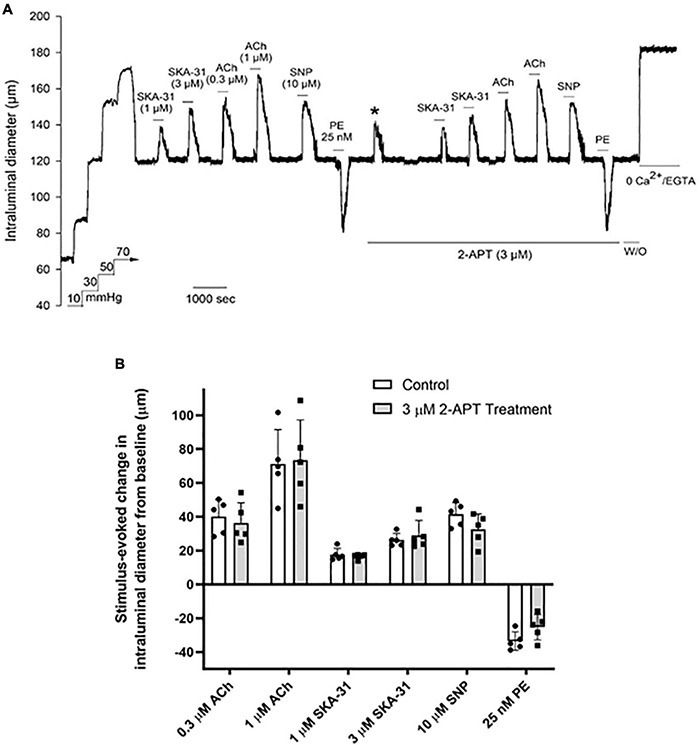
The selective NOX1 inhibitor 2-APT does not modify vasoactive responses in cremaster arteries from adult Sprague–Dawley rats. The representative tracing in panel **(A)** shows the effects of endothelium-dependent and -independent vasoactive agents in the absence and presence of 3 μM 2-acetylphenothiazine (2-APT). Compounds were present in the bath as indicated by the horizontal bars above and below the tracing. The asterisk above the tracing denotes a transient dilatory response that was typically observed following bath addition of 3 μM 2-APT. Maximal arterial diameter was determined at the end of the protocol by addition of nominally Ca^2+^ free physiological saline solution (i.e., 0 added CaCl_2_ + 2 mM EGTA). The histogram in panel **(B)** quantifies the absolute changes in intraluminal diameter evoked by each vasoactive agent in the absence and presence of 3 μM 2-APT treatment (*n* = 5). Data are presented as means ± S.D.

To interrogate the potential impact of NADPH oxidase inhibition on vasoactive responses to endothelium-dependent and –independent agents, we examined the vasomotor effects of ACh, SNP, PE and the endothelial KCa channel activator SKA-31 (Sankaranarayanan et al., 2009) in intact, myogenically contracted cremaster arteries. As shown in Figure 4A, treatment with the NOX1 selective inhibitor 2-APT (3 μM) did not alter vasoactive responsiveness to these exogenous stimuli, which is further confirmed by quantification of individual responses observed in the absence and presence of 2-APT (Figure 4B). Our biochemical data indicate that the selected concentration of 2-APT (i.e., 3 μM) inhibited total NADPH oxidase activity in aortic tissue homogenates by 86% (Table 2). Functionally, exposure to 3 μM 2-APT caused a transient inhibition of basal myogenic tone that reversed within 10 min (denoted in Figure 4A by the asterisk above the dilatory response at the start of 2-APT addition). This transient vasodilatory effect was commonly observed at 2-APT concentrations < 30 μM, as illustrated in Figure 2A. We were unable to examine the effects of VAS2870 treatment on endothelium-dependent and -independent vasoactive stimuli, given its robust inhibition of basal myogenic tone at concentrations that produce meaningful inhibition of vascular NADPH oxidase activity (see Figure 2B).

### The Non-selective NOX Inhibitor Apocynin Does Not Modify Vasoactive Responses in Cremaster Arteries

**FIGURE 5:**
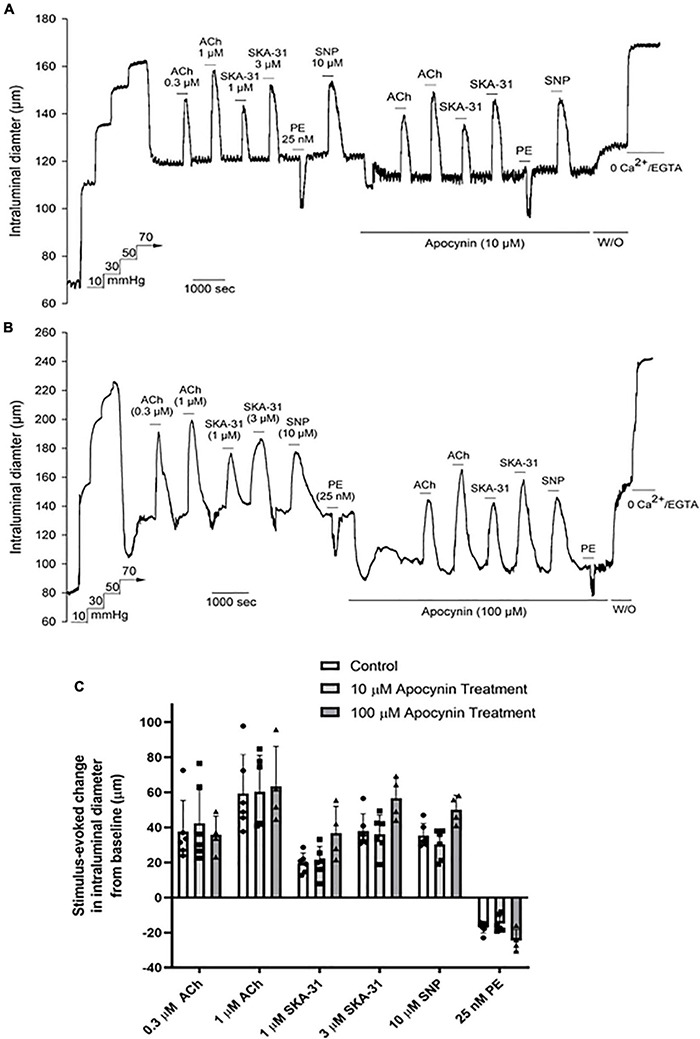
The non-selective NOX inhibitor apocynin has minimal effects on vasoactive responses in Sprague–Dawley rat cremaster arteries. The representative tracings in panels **(A,B)** show the effects of endothelium-dependent and -independent vasoactive agents in the absence and presence of 10 μM and 100 μM apocynin treatment, respectively. Agents were present in the bath solution as indicated by the horizontal bars above and below the tracings. Maximal arterial diameter was determined at the end of the protocol by addition of nominally Ca^2+^ free physiological saline solution (i.e., 0 added CaCl_2_ + 2 mM EGTA). The histogram in panel **(C)** quantifies the absolute changes in intraluminal diameter evoked by each vasoactive agent in the absence and presence of 10 μM and 100 μM apocynin treatment (*n* = 8 arteries and 4 arteries, respectively). Data are presented as means ± S.D. No statistically significant differences from control responses were noted, as determined by a one-way ANOVA and a Newman–Keuls *post hoc* test.

As treatment with 3 μM 2-APT may not effectively inhibit NOX isoforms 2–4, given the reported IC_50_ values of 2-APT for these enzymes (Selemidis et al., 2008; Altenhöfer et al., 2015), we examined the effects of the non-selective NADPH oxidase inhibitor apocynin (10 and 100 μM) on vasoactive responses to ACh, SNP, PE and SKA-31 in myogenically active cremaster arteries. Parallel biochemical assays revealed that apocynin at these concentrations inhibited total NADPH oxidase activity in aortic tissue homogenates by 49% and 88%, respectively (Table 2). Apocynin is also reported to exhibit anti-oxidant behavior at similar concentrations (Heumuller et al., 2008). As shown by the representative tracings in Figures 5A,B, apocynin treatment alone evoked additional constriction in myogenically active vessels, leading to a greater reduction in intraluminal diameter that reversed upon washout. Interestingly, this observed increase in contractility was opposite to the effects of 2-APT and VAS2870 on basal myogenic tone (Figure 2A). Despite the increased contractile tone, treatment with apocynin at either 10 or 100 μM did not modify vasoactive responses to either endothelium-dependent or –independent agents, as quantified in Figure 5C. Collectively, the data presented in Figures 4, 5 indicate that treatment of myogenically active cremaster arteries with the NOX inhibitors 2-APT and apocynin did not modify acute vasoactive responses to endothelium-dependent and –independent agents, despite having pronounced inhibitory effects on vascular NADPH oxidase activity at the same experimental concentrations.

### Vasoactive Responses in Cremaster Arteries From Type 2 Diabetic Rats Are Unaffected in the Presence of Either 2-APT or Apocynin

**FIGURE 6:**
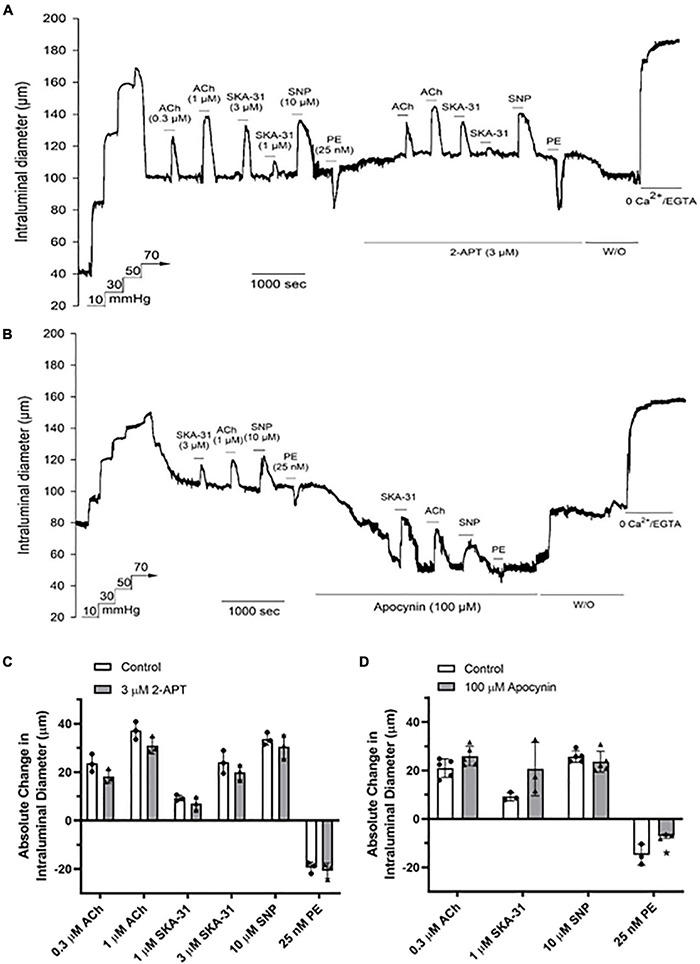
The NOX inhibitors 2-APT and apocynin have minimal effects on vasoactive responses in myogenically active cremaster arteries from T2D Goto-Kakizaki rats. The representative tracings in panels **(A,B)** show the effects of endothelium-dependent and -independent vasoactive agents in the absence and presence of 3 μM 2-APT and 100 μM apocynin treatment, respectively. Compounds were added to the bath as indicated by the horizontal bars above and below the tracings. Maximal arterial diameter was determined at the end of the protocol by addition of nominally Ca^2+^ free physiological saline solution (i.e., 0 added CaCl_2_ + 2 mM EGTA). The histograms in panels **(C,D)** quantify the absolute changes in intraluminal diameter evoked by each vasoactive agent in the absence and presence of 2-APT (*n* = 3 animals) or apocynin (*n* = 3–5). The asterisk indicates a statistically significant change from the control response, as determined using a two-tailed Student’s *t*-test; *P* < 0.05. Data are presented as means ± S.D.

Several studies have reported that reactive oxygen species and/or H_2_O_2_ may contribute to vasodilatory signaling under conditions of endothelial dysfunction (i.e., reduced NO bioavailability) (Liu et al., 2003, 2011; Miura et al., 2003; Ellinsworth et al., 2015; Gutterman et al., 2016). Cremaster resistance arteries from spontaneous Type 2 Diabetic Goto-Kakizaki (T2D GK) rats exhibit endothelial dysfunction and impaired vasodilatory signaling that are consistent with decreased NO availability (Mishra et al., 2021). To determine the impact of NOX inhibition on vasoactive signaling under conditions of endothelial dysfunction, we examined vasodilatory and vasoconstrictor responses in cremaster resistance arteries from adult T2D GK rats. As shown in Figure 6A, treatment with 3 μM 2-APT caused a modest decrease of basal myogenic tone (control = 38.3 ± 4.2% vs. 32.1 ± 5.7% in the presence of 2-APT, *n* = 3), but did not alter the magnitude of vasoactive responses to stimuli acting *via* the endothelium or vascular smooth muscle (Figure 6C). In contrast, exposure of T2D GK cremaster arteries to 100 μM apocynin produced a substantial and reversible constriction of intraluminal diameter (control = 38.1 ± 3.9% vs. 54.6 ± 7.1% in the presence of apocynin, mean ± SD, *n* = 5, *P* = 0.002) (Figure 6B), but did not modify vasodilation in response to either endothelium-dependent or –independent agents (Figure 6D). However, the PE-induced contractile response was dampened in the presence of apocynin.

### The Transient Vasodilatory Actions of 2-APT Are Minimally Affected by Pharmacological Inhibition of Endothelium-Mediated Vasodilatory Signaling

**FIGURE 7:**
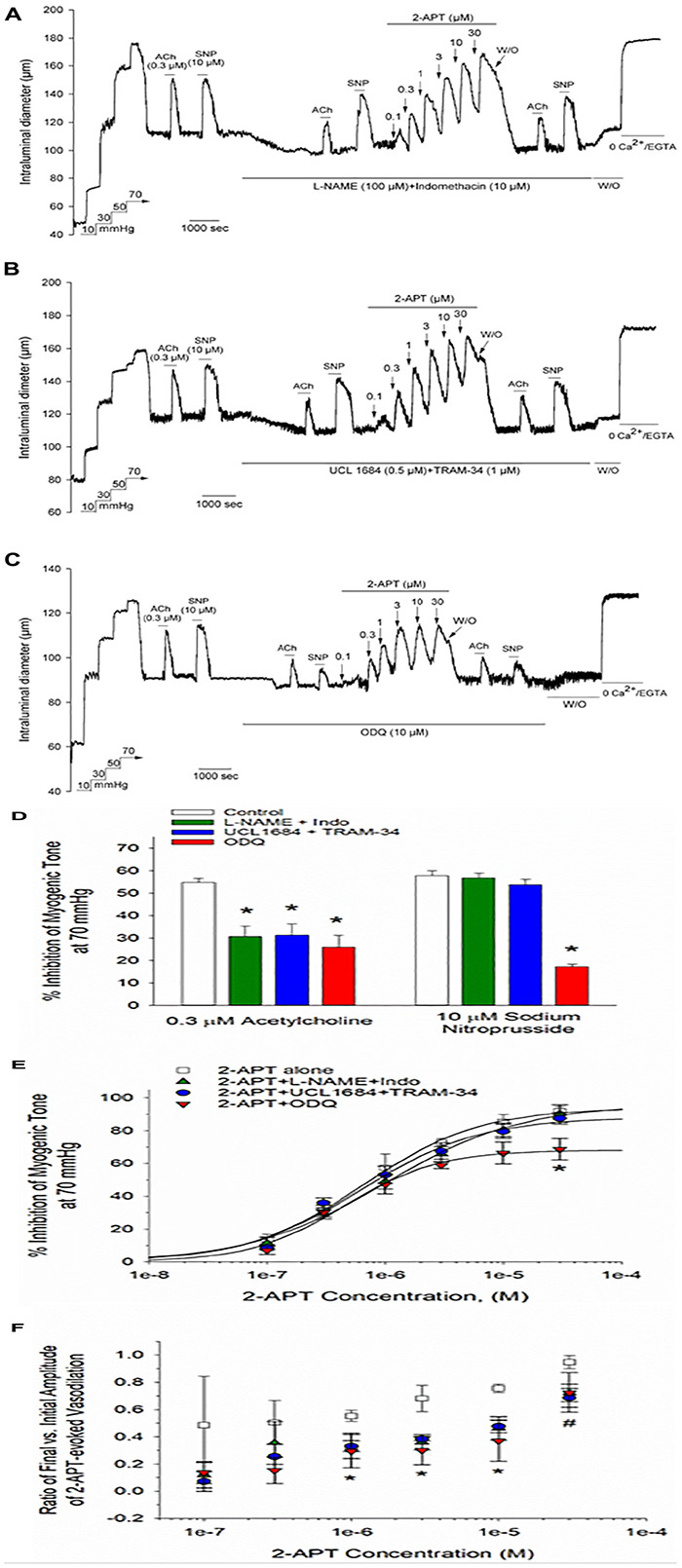
Acute inhibition of established vasodilatory signaling pathways does not impair the 2-APT-evoked vasodilation of Sprague–Dawley rat cremaster arteries. The representative tracings in panels **(A–C)** depict the concentration-dependent vasodilation of myogenically constricted cremaster arteries by increasing concentrations of 2-APT in the presence of **(A)** L-NAME (100 μM) + Indomethacin (10 μM), **(B)** UCL 1684 (0.5 μM) + TRAM-34 (1 μM), inhibitors of endothelial KCa2.3 and KCa3.1 channels, respectively, or **(C)** the soluble guanylyl cyclase inhibitor ODQ (10 μM). Under each treatment condition, arteries were also stimulated with the endothelium-dependent vasodilator acetylcholine (ACh, 0.3 μM) and the smooth muscle relaxant sodium nitroprusside (SNP, 10 μM) to verify the effectiveness of each treatment. The horizontal lines above and below the tracing indicate the presence of the indicated stimulus or treatment. The histogram in panel **(D)** quantifies ACh and SNP evoked vasodilation in the absence and presence of each treatment. The plot in panel **(E)** quantifies the peak inhibition of myogenic tone at each concentration of 2-APT under each of the treatment conditions described above. The solid line fits to the data points were calculated using a Hill equation (see section “Materials and Methods”). The plot in panel **(F)** describes the ratio of the evoked 2-APT response remaining after 8 min treatment vs. the peak inhibitory response observed at ∼1 min for each 2-APT concentration in the presence of the noted treatment conditions. A ratio of 1 would indicate no decay in the magnitude of response over time. Ratios for the magnitude of 2-APT evoked dilation observed at the end vs. the beginning of the 8 min treatment period under control conditions were calculated from tracings equivalent to that presented in Figure 2A. Data are presented as mean ± S.D. (*n* = 13 arteries for the control condition, and 3 for each of the described treatment conditions). Statistical analysis was performed using a one-way ANOVA and a Newman–Keuls *post hoc* test. An asterisk indicates a significant difference from the control value, *P* < 0.05. The # sign denotes a statistical difference vs. control for 2-APT evoked responses in the presence of either L-NAME + Indomethacin or UCL 1684 + TRAM-34, *P* < 0.05.

In view of the unexpected and robust vasodilatory action of 2-APT on myogenically constricted cremaster arteries (see Figure 2), we explored the potential underlying cellular mechanism by examining 2-APT effects in the absence and presence of established inhibitors of prominent endothelial-signaling pathways. Vasodilatory responses to ACh and SNP were utilized as internal controls to verify the effectiveness of individual treatment protocols on targeted cellular pathways (Figure 7D). As shown in Figure 7A, inhibition of NO synthase/cyclo-oxygenase signaling by L-NAME + indomethacin did not noticeably alter either the magnitude or sensitivity of 2-APT-evoked vasodilation in intact cremaster arteries from adult Sprague–Dawley rats. A similar result was observed for 2-APT action in the presence of UCL-1684 and TRAM-34, selective inhibitors of KCa2.3 and KCa3.1 channels that mediate endothelium-dependent hyperpolarization (Félétou, 2009; Köhler et al., 2016; Khaddaj Mallat et al., 2017; Figure 7B). However, exposure of cremaster arteries to the guanylyl cyclase inhibitor ODQ (10 μM) significantly reduced the magnitude of 2-APT evoked vasodilation (Figure 7C). Figure 7E quantifies the effects of all three treatment conditions on the extent and sensitivity of 2-APT induced vasodilation.

Although inhibition of endothelial and smooth muscle signaling pathways had either modest or no effects on the magnitude of 2-APT evoked vasodilation, it was evident from the original tracings that the vasodilatory responses to 2-APT decayed more rapidly under these treatment conditions. By calculating the fraction of peak 2-APT evoked dilation remaining following ∼8 min of 2-APT exposure, we found that all the treatments described above hastened the recovery of myogenic contraction to baseline levels in the continued presence of 2-APT, which was most prominent for 2-APT concentrations > 1 μM (Figure 7F). Exactly how these varied treatments promoted this decay or “desensitization” of the 2-APT mediated vasodilation remains unclear.

## Discussion

H_2_O_2_ is reported to have vasoactive effects in a variety of arterial preparations (Matoba et al., 2000, 2002; Miura et al., 2003; Thengchaisri and Kuo, 2003; Cseko et al., 2004; Li et al., 2016) and to serve as an alternate vasodilatory mediator under pathophysiological conditions in which NO bioavailability is compromised (Liu et al., 2003, 2011; Miura et al., 2003; Ellinsworth et al., 2015; Gutterman et al., 2016). Physiologically, H_2_O_2_ production has been detected in cell types known to regulate vascular tone, such as endothelium, smooth muscle and nerves, which further supports its putative role as an endogenous vasodilatory compound. In the present study, we sought to examine the potential contribution of H_2_O_2_ to the vasoactive effects of endothelium-dependent and –independent agents in myogenically constricted rat skeletal muscle arteries by utilizing reported pharmacological inhibitors of NADPH oxidase enzymes to restrict acute H_2_O_2_ generation.

Myogenically constricted cremaster arteries from adult Sprague–Dawley rats exhibited robust vasodilation in response to bath applied H_2_O_2_ at concentrations > 10 μM (Figure 1), which is similar to responses observed in other myogenically constricted arteries (Thengchaisri and Kuo, 2003; Cseko et al., 2004; Lucchesi et al., 2005; Li et al., 2016). This observed sensitivity to exogenous H_2_O_2_ thus suggests that myogenically active arteries are capable of responding to endogenously produced H_2_O_2_.

**FIGURE 8:**
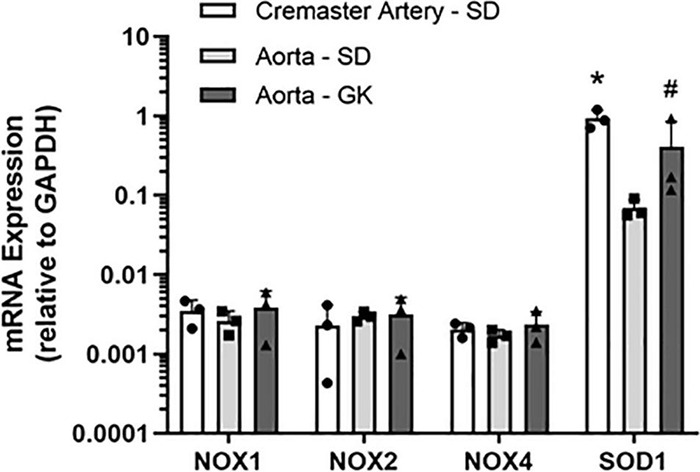
Relative expression of mRNA encoding NADPH oxidase (NOX) isoforms in Sprague–Dawley (SD) and Goto-Kakizaki (GK) rat cremaster arteries and aorta. The expression levels of NOX1, 2 and 4 isoforms in each vessel type were quantified by qRT-PCR and are presented as ratios to the expression of GAPDH mRNA in the same vessel type. Measurements were performed in tissues from 3 individual animals. Statistical analysis was performed using a one-way ANOVA and a Newman–Keuls *post hoc* test. The asterisk denotes a statistically significant difference relative to SD aorta, *P* < 0.01; the # symbol indicates a difference vs. SD aorta; *P* < 0.05. Data are presented as means ± S.D.

Using an independent biochemical assay, we observed that reported pharmacological inhibitors selective for NOX 1 (i.e., 2-APT) and NOX 2 (i.e., VAS2870), along with the non-selective NOX inhibitor apocynin, inhibited vascular NADPH oxidase activity by > 85% in a concentration-dependent manner (Table 2). These data demonstrate the efficacy of these inhibitors and are supported by our quantitative detection of mRNA for NOX1, NOX2 and NOX4 in aorta and cremaster arteries (Figure 8). These observations further agree with more detailed reports describing the expression of NOX isozymes in vascular smooth muscle and endothelium (Bedard and Krause, 2007; Selemidis et al., 2008). Due to the limited availability of small resistance arteries for mRNA isolation, particularly from T2D Goto-Kakizaki rats, we were unable to screen all vessel types used in our study for the expression of NOX isozymes.

Treatment of myogenically constricted arteries from adult Sprague–Dawley rats with either 100 μM apocynin or 3 μM 2-APT, concentrations that produce > 85% inhibition of vascular NADPH oxidase activity (Table 2), did not modify stimulus-evoked vasoactive responses acting *via* the endothelium (i.e., ACh, SKA-31) or smooth muscle (i.e., SNP, phenylephrine) (Figures 4, 5). These observations suggest that acute generation of superoxide, and presumably H_2_O_2_, *via* NADPH oxidase does not contribute to the vasoactive signaling pathways stimulated by these agents in myogenically constricted resistance arteries from healthy rats. Similarly, we did not observe any significant effects of either 2-APT or apocynin on vasodilatory responses in cremaster skeletal muscle arteries from adult T2D Goto-Kakizaki rats, which exhibit endothelial dysfunction (Mishra et al., 2021; Figure 6). Bitar et al. (2005) have reported that superoxide production and NADPH oxidase subunit expression are elevated in aortae from T2D Goto-Kakizaki rats; these factors would be expected to contribute to endothelial dysfunction in these animals by decreasing NO bioavailability. Our findings from T2D resistance arteries thus contrast data showing that H_2_O_2_ may serve as a vasodilatory signaling molecule under pathophysiological conditions involving compromised NO-mediated dilation, as reported in small arteries obtained from patients undergoing coronary artery bypass or cardiac valve replacement surgery (Liu et al., 2003, 2011; Miura et al., 2003; Phillips et al., 2007). It is possible that the vascular bed, species or type of stimulus acting on the endothelium (e.g., receptor agonist vs. flow-induced shear stress) could account for such differences. We further recognize that the limited sample sizes in some of these protocols (e.g., *N* values = 3–4) may lead to a Type II statistical error (i.e., a false negative) that could obscure potential differences in the magnitude of response.

A major, but unexpected finding in our study is that the NADPH oxidase inhibitors we utilized had direct and substantive effects on basal myogenic contractility in resistance arteries from two distinct vascular beds (i.e., skeletal muscle and cerebral circulation). Whereas apocynin treatment promoted vasoconstriction, 2-APT and VAS2870 evoked near-maximal dilation of myogenically active cremaster arteries in a concentration-dependent manner (Figures 2, 5). The similar efficacy of VAS2870 in both intact and endothelium-denuded cremaster and middle cerebral arteries strongly suggest that the vasodilatory action of this compound occurs largely *via* the vascular smooth muscle. In contrast, 2-APT-evoked vasodilation was blunted substantially in denuded vs. intact cremaster arteries, indicating an important role for the endothelium. In intact middle cerebral arteries, bath applied 2-APT evoked only a modest vasodilatory response compared with cremaster arteries (see Figures 2, 3), whereas intraluminal application of 2-APT in cerebral vessels produced a significantly larger vasodilatory response (Supplementary Figure 1). Taken together, these data suggest that extraluminal 2-APT acts primarily on the outer smooth muscle layer in middle cerebral arteries and may not readily penetrate the internal elastic lamina to reach the endothelium. These observations are thus reminiscent of our earlier results showing that bath-applied ACh evokes a weak and inconsistent vasodilation in rat middle cerebral arteries (Mishra et al., 2015).

In contrast to 2-APT, we observed that the selective NOX2 agent VAS2870 inhibited myogenic contraction in a largely irreversible manner in both cremaster and middle cerebral arteries, as evidenced by the maintained loss of myogenic tone following VAS2870 washout (Figures 2B,C, 3A,B). Furthermore, contractile responses to both a GPCR-mediated (i.e., phenylephrine) and depolarization-dependent (i.e., elevated external KCl) vasoconstrictor were largely abolished following VAS2870 treatment and washout. Exactly how VAS2870 treatment leads to long-term modification of vascular smooth muscle to impair these diverse contractile stimuli in the absence of VAS2870 is unclear, and may involve one or more off-target effects (e.g., contractile machinery, Ca^2+^ dynamics/signaling, ion channel activity). Other investigators have reported that VAS2870 inhibits cellular function independent of NOX activity (Gatto et al., 2013; Lu et al., 2019) and may interfere with protein kinase C (PKC) signaling. As PKC activity has been implicated in myogenic contractility (Earley et al., 2007; Schubert et al., 2008; Moreno-Dominguez et al., 2014), such inhibition may contribute to the negative effects of VAS2870 observed here. To the best of our knowledge, this is the first study to report such results in myogenically active resistance arteries.

Our results demonstrating that acute treatment of myogenically constricted arteries with apocynin, 2-APT and VAS2870 produced discordant effects (i.e., vasoconstriction vs. vasodilation, reversible vs. irreversible), despite their common inhibitory effects on vascular NADPH oxidase activity, indicate that these chemically diverse compounds likely have independent, off-targets effects on the myogenic contractile machinery in small resistance arteries. Importantly, these non-specific effects occurred over the same concentration range described for their enzymatic inhibition of cellular NADPH oxidase. While it is possible that the observed effects of these compounds on myogenic contractility could be linked to acute inhibition of endogenous superoxide production in myogenically active arteries, we believe that this likelihood is low, given the disparate actions of these agents on basal myogenic tone, as highlighted in Supplementary Figure 2.

While the irreversible inhibition of myogenic tone by VAS2870 in cremaster and cerebral arteries precluded any further investigation with this compound, we explored the possible cellular mechanism(s) underlying the vasodilatory actions observed for 2-APT (see Figures 2, 3). In intact cremaster arteries, 2-APT evoked a robust vasodilation that decayed over time (Figures 2A, 4A). In contrast, the magnitude of the 2-APT evoked dilation was decreased substantially in endothelium-denuded cremaster arteries, and responses exhibited minimal decay over time, as shown in Figure 2C. Investigation of the putative signaling pathway(s) underlying the endothelium-dependent, vasodilatory actions of 2-APT revealed that pharmacological blockade of eNOS and cyclo-oxygenase signaling with L-NAME + indomethacin, or endothelium-dependent hyperpolarization using blockers of KCa2.x and KCa3.1 channels (i.e., UCL 1684 + TRAM-34) did not modify either the peak vasodilatory response or potency of 2-APT. However, inhibition of soluble guanylyl cyclase activity with ODQ modestly reduced the magnitude of 2-APT evoked vasodilation (Figure 7E), suggesting that a portion of the 2-APT effect is mediated by cGMP signaling in vascular smooth muscle. Another possibility is that the redox capability of ODQ may lead to chemical modification/inactivation of 2-APT at concentrations ≥ 10 μM. Inhibition of these vasodilatory pathways in the vascular wall increased the transient nature of the 2-APT responses, resulting in an apparent “desensitization” (Figure 7F). The cellular mechanism(s) underlying this 2-APT evoked vasodilation and its transient behavior remain unclear.

In summary, we have observed that reported inhibitors of NADPH oxidase enzymes do not alter agonist-evoked, endothelium-dependent vasodilatory responses in skeletal muscle resistance arteries from either healthy or T2D adult male rats. Our data further reveal that these compounds can alter myogenic contractility in small resistance arteries in a manner that is difficult to reconcile with respect to acute changes in superoxide and/or H_2_O_2_ availability. These putative off-target effects raise questions about the use of such agents in intact cardiovascular tissues and emphasize the importance of rigorous control data.

## Data Availability Statement

The original contributions presented in the study are included in the article/Supplementary Material, further inquiries can be directed to the corresponding author/s.

## Ethics Statement

The animal study was reviewed and approved by University of Calgary Animal Care Committee.

## Author Contributions

DK, RM, CJ, and H-LZ: investigation, methodology, formal analysis, and data presentation. AB: conceptualization, formal analysis and data presentation, writing-original draft, writing-review and editing, supervision, project administration, and funding acquisition. All authors contributed to final approval of the submitted version of the manuscript.

## Conflict of Interest

The authors declare that the research was conducted in the absence of any commercial or financial relationships that could be construed as a potential conflict of interest.

## Publisher’s Note

All claims expressed in this article are solely those of the authors and do not necessarily represent those of their affiliated organizations, or those of the publisher, the editors and the reviewers. Any product that may be evaluated in this article, or claim that may be made by its manufacturer, is not guaranteed or endorsed by the publisher.
