# Supplementary material for: Effects of Pharmacological Inhibitors of NADPH Oxidase on Myogenic Contractility and Evoked Vasoactive Responses in Rat Resistance Arteries
Source: Front Physiol. 2022 Jan 24;12:752366. doi: 10.3389/fphys.2021.752366 (PMC8818784; doi:10.3389/fphys.2021.752366)
Supplement: Supplementary file 1 [file Data_Sheet_1.pdf]

Supplementary Information

Supplementary Figure 1 – Intraluminal application of 2-APT evokes greater dilatory responses in intact cerebral arteries vs. bath perfusion. The representative tracing in panel A displays a myogenically active middle cerebral artery with continuous intraluminal perfusion at a rate of ~25 μl/min. Bath application of 3 μM SKA-31 and 10 μM 2-APT was followed by intraluminal application of 10 μM 2-APT, as indicated. The horizontal bars above the recording denote the timing and duration of each drug application. Following washout of 2-APT from the vessel lumen, bath application of SKA-31 was repeated to verify the functional integrity of the endothelium. Maximal arterial diameter was determined at the end of the protocol by addition of nominally Ca^{2+} free physiological saline solution (i.e. 0 added $CaCl_2$ + 2 mM EGTA). The histogram in panel B quantifies the percent inhibition of myogenic tone evoked by bath-applied 2-APT vs. intraluminal application, along with the inhibition of myogenic tone evoked by bath-applied 3 μM SKA-31 prior to and following 2-APT administrations. The asterisk indicates a statistically significant difference vs. bath-applied 2-APT, as determined by an unpaired Student's t-test, $P < 0.05$. Data are presented as means ± S.D. (n = 3).

Supplementary Figure 2 – Relative effects of NOX inhibitors on basal myogenic tone in cremaster arteries from Sprague Dawley rats vs. inhibition of aortic tissue NADPH oxidase activity. The bars describe the level of steady-state myogenic constriction recorded at 70 mmHg intraluminal pressure in either the absence (i.e. control) or presence of the indicated concentrations of NOX inhibitors. Open circles represent individual arteries under each experimental condition. The red squares plot the level of NADPH oxidase inhibition produced by each NOX inhibitor in aortic tissue homogenates (data reproduced from Table 2). This dual plot highlights the discordant effects of these NOX inhibitors on myogenic contractility vs. vascular NADPH oxidase activity. The asterisk indicates a statistically significant difference vs. basal myogenic tone under control conditions, as determined by a one-way ANOVA and a Newman-Keuls post-hoc test, $P < 0.05$.
