# Supplementary figures and images for: Effects of Pharmacological Inhibitors of NADPH Oxidase on Myogenic Contractility and Evoked Vasoactive Responses in Rat Resistance Arteries
Source: Front Physiol. 2022 Jan 24;12:752366. doi: 10.3389/fphys.2021.752366 (PMC8818784; doi:10.3389/fphys.2021.752366)

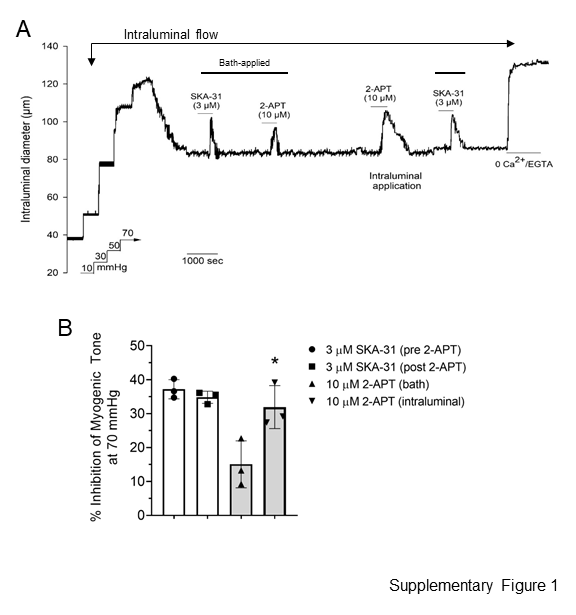

Supplement: Supplementary file 2 [file Image_1.tif]

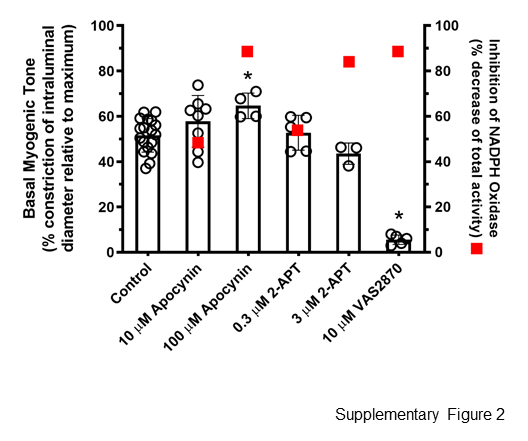

Supplement: Supplementary file 3 [file Image_2.tif]
